# Antidepressant medication use and prostate cancer recurrence in men with depressive disorders

**DOI:** 10.1007/s10552-022-01623-5

**Published:** 2022-09-09

**Authors:** Reina Haque, Stephanie Reading, Michael R. Irwin, Lie Hong Chen, Jeff Slezak

**Affiliations:** 1 Department of Research & Evaluation, Kaiser Permanente Southern California, 100 South Los Robles, 2nd Floor, Pasadena, CA 91101 USA; 2 Department of Health Systems Science, Kaiser Permanente Bernard J. Tyson School of Medicine, Pasadena, CA 91101 USA; 3 Department of Psychiatry and Biobehavioral Sciences, Geffen School of Medicine, University of California at Los Angeles, Los Angeles, CA 90095 USA

**Keywords:** Prostate cancer, Depression, Recurrence, Antidepressants

## Abstract

**Purpose:**

Whether treating prostate cancer survivors with a depressive disorder with antidepressants can affect their cancer outcomes is unknown. We evaluated the association between antidepressant use and prostate cancer recurrence, in survivors with comorbid depressive disorders.

**Methods:**

We conducted a longitudinal cohort study of 10,017 men with prostate cancer (stages I–II) diagnosed who also had a comorbid depressive disorder followed a maximum of 22 years, and examined rates of biochemical recurrence by antidepressant medication use. We conducted multivariable Cox models based on time-dependent antidepressant drug use status, and examined the risk of biochemical recurrence by cumulative duration of antidepressant use.

**Results:**

Of these 10,017 survivors, 1842 (18%) experienced biochemical recurrence over 69,500 person-years of follow-up. The prostate cancer biochemical recurrence rate was greater with antidepressant non-use (31.3/1000 person-years) compared to antidepressant use (23.5/1000 person-years). In Cox proportional hazards multivariable adjusted models, non-use of antidepressants was associated with a 34% increased risk of biochemical recurrence compared to antidepressant use (HR = 1.34, 95% CI: 1.24–1.44). Longer use of antidepressants was associated with a lower biochemical recurrence risk (*P* trend test < 0.001).

**Conclusion:**

Untreated depressive disorders in prostate cancer patients may be associated with an increased risk of biochemical recurrence.

## Introduction

Nearly 200,000 men are diagnosed with prostate cancer annually in the USA, and the population of survivors has surpassed 3 million [1, 2]. In prostate cancer survivors, depression prevalence is about threefold higher as compared to the general community of men [3–5] and depression is thought to contribute to adverse cancer outcomes. Indeed, one study found that depression history predicted increased mortality in prostate cancer survivors regardless of whether the depression diagnosis was made before or after cancer diagnosis and treatment [6]. In addition, in those with other cancers, depression and depressive symptoms are associated with worse outcomes such as higher cancer recurrence rates, more comorbidities, and a higher mortality risk than those without depression [7–9].

Less is known if antidepressant treatment can mitigate these risks. In women with metastatic breast cancer, antidepressant treatment was found to reduce depressive symptom severity over the first year after cancer diagnosis, and improvements in depression were associated with longer survival [9]. In 25 prostate cancer survivors, one small clinical trial found that antidepressant medication treatment with monoamine oxidase inhibitor (MAOI) decreased prostate specific antigen (PSA) levels 12 weeks after therapy; however, that study could not ascertain recurrence risk due to its short follow-up [10]. In sum, no large population-based studies have specifically evaluated if antidepressant use can reduce biochemical recurrence risk in prostate cancer survivors with comorbid depressive disorders.

As antidepressants remain the cornerstone of depression treatment [11, 12], the objective of this cohort study was to evaluate the association between reported antidepressant medication use and recurrence of prostate cancer as defined by American Urological Association (AUA) guidelines in a sample of 10,017 prostate cancer survivors with a comorbid depressive disorder. In addition, we evaluated if cumulative duration of antidepressant use was associated with a lower risk of recurrence.

## Methods

### Study design, subjects and setting

Subjects were identified from the Kaiser Permanente Southern California (KPSC) health plan, a not-for-profit integrated healthcare delivery system comprised 15 community hospitals, over 220 medical offices and serving more than 4.7 million members. Patients receive virtually all of their medical care, including pharmacy prescriptions, within this system and medical procedures and diagnoses outside of the system are captured from claims databases. Patients were identified using the health plan’s U.S. National Cancer Institute’s Surveillance Endpoints & End Results (SEER)-affiliated cancer registry.

The inclusion criteria for this longitudinal cohort study included: (1) all adult men (≥ 18 years) newly diagnosed with early stage prostate cancer (American Joint Committee on Cancer [AJCC] TNM stages I-II) from January 1996 to June 2017 (*n =* 36,335), and (2) those with an documented comorbid depressive disorder in their electronic health records (EHR), inclusive of year prior to their prostate cancer diagnosis through end of follow-up, and (3) at least six months of follow-up post cancer diagnosis. A combination of three data sources were used to identify the earliest documented depressive disorder; and the depression must have appeared in two of the three data sources. If dates of the depressive disorder were different in these data sources, we used the earliest date. The three data sources were: (1) having an inpatient or outpatient diagnostic code for a depressive disorder in the patient’s EHR (ICD-9-Clinical Modification [ICD-9-CM] 296.2, 296.3, 296.5, 296.6, 296.7, 298.0, 301.10, 301.12, 301.13, 309.0, 309.1, 311 [13]) – ICD-9-CM codes from the inpatient database were prioritized over outpatient database; (2) manual review of the current problem list within the patients’ EHR; and (3) manual review of documentation of a depressive disorder from a natural language-assisted review of clinicians’ notes within the patients’ EHR. We prioritized the information garnered from chart review of clinicians’ notes, then the problem lists. No other exclusions were applied. A total of *n =* 10,017 men fit the criteria, and we followed them through study’s end, 31 December 2018 (22 years maximum follow-up).

### Prostate cancer biochemical recurrence

The main outcome, prostate cancer biochemical recurrence was based on the definitions of the American Urological Association (AUA) and the American Society for Therapeutic Radiology & Oncology (ASTRO) guidelines [14]. Briefly, these specifications define biochemical recurrence as occurring at least six months following the initial early stage prostate cancer diagnosis. This six-month window is based on the standard definitions of biochemical recurrence with prostate-specific antigen (PSA) data, which published reports agree are reliable in detecting early disease recurrence [15, 16]. Using this six-month window, the definition is further divided by type of prostate cancer treatment the patient receives. These treatment-based definitions are as follows [15]. For those who underwent radical prostatectomy, biochemical recurrence was defined as: (1) two consecutive PSA values ≥ 0.2 ng/ml at least six months after initial surgery date, or (2) a second treatment initiated six months or more after initial surgery date. For those who underwent radiation therapy (external beam or brachytherapy), biochemical recurrence was defined as either: (1) a rise in PSA by ≥ 2 ng/ml above the PSA nadir, or (2) a second treatment initiated six months or more after the initial radiation therapy. For those who received hormonal therapy, biochemical recurrence was defined as either: (1) three consecutive rising PSA values after initiation of hormonal therapy, (2) or a second treatment initiated six months or more after the initial hormonal therapy. For those who elected active surveillance or watchful waiting, which we defined as initiating no treatment within one year of the patient’s prostate cancer diagnosis, prostate cancer biochemical recurrence was defined as either: (1) three consecutive rising PSA values starting at least one year after diagnosis, or (2) a single value of at least 2 ng/mL above the PSA level at the time of diagnosis at least one year after diagnosis, or (3) initiation of treatment six months or more after the initial prostate cancer diagnosis. We used the earliest identified outcome as the date of the biochemical recurrence.

### Antidepressant use

We examined antidepressant use starting from the drug’s date of initiation post prostate cancer diagnosis until the earliest of one of the study endpoints (biochemical recurrence; disenrollment from health plan; death; study’s end in December 2018). Antidepressant use was extracted from the health plan’s pharmacy dispensing records; data elements included the drug names, initiation date, and days supplied. The study antidepressants were the following: selective serotonin reuptake inhibitors (SSRI); serotonin antagonist and reuptake inhibitors (SARIs); serotonin modulator and stimulators (SMS); serotonin modulator and stimulators (SNRI); monoamine oxidase inhibitors (MOAIs); norepinephrine-dopamine reuptake inhibitors (NRI, NDRI); and tricyclic and tetracyclic antidepressants (TCA, TECA). We also searched for psychotherapy CPT-4 codes in the administrative and claims databases (e.g., 90832, 90834, 90837, 90838); however, we found few instances of psychotherapy (talk therapy) in the EHR.

### Covariates

We captured the following covariates: age; stage; and year of prostate cancer diagnosis; baseline PSA; Gleason score; race/ethnicity; geocoded median household income (based on 2010 U.S. Census at the block level); body mass index (kg/m^2^) closest to prostate cancer diagnosis; smoking and alcohol misuse during follow-up (ICD-9-CM codes: 303.90–93 and 305.00–305.03); Charlson Comorbidity Index in the one-year before prostate cancer diagnosis using the Deyo method [16, 17]; anxiety disorder history (ICD-9-CM 293.84, 300.0, 300.01, 300.02, 300.09, 308); anti-anxiety medication use (alprazolam; chlordiazepoxide; clonazepam; diazepam; lorazepam); and prostate cancer treatment type [radiotherapy; hormonal (androgen deprivation therapy); watchful waiting/active surveillance; surgery (prostatectomy). Statin and metformin use during follow-up were also captured. We also accounted for annualized outpatient office visits because patients with more clinic visits might have had a greater likelihood of being diagnosed with biochemical recurrence or depressive disorders.

### Statistical analysis

In descriptive analyses, we examined the frequencies and proportions of all covariates initially by ever antidepressant use. *P*-values for assessing the differences were conducted using Chi-square tests for categorical variables and the Wilcoxon-Mann–Whitney test for continuous variables. Due to the varying lengths of patients’ follow-up time, we computed incidence rates of biochemical recurrence by antidepressant use status. Crude and adjusted hazards ratios and corresponding 95% confidence intervals for biochemical recurrence risk were computed using Cox proportional hazard models, and antidepressant use was treated as time-dependent variables in the multivariable models. In these analyses, patients’ antidepressant use was tracked starting from date of drug initiation after prostate cancer diagnosis until the earliest study endpoint. The multivariable models accounted for the aforementioned covariates including sociodemographics, year of diagnosis, hospital, tumor stage, tumor characteristics and treatment, ADT, comorbidity status, smoking and alcohol misuse, anxiety, antipsychotic medication use, use of statin and metformin, annualized number of outpatient visits, and hospital. All medications, including ADT, we handled as time-varying variables in the multivariable models. Final models were selected based on the combination of goodness of fit, assessment of collinearity among covariates, and clinical factors associated with both biochemical recurrence and antidepressant use. There was a relatively low percentage of missing values of the covariates, therefore, missing values (< 2% for most variables) were handled as an additional category in all models. This was decided based on a complete-case assessment, restricting the analysis to individuals with no missing covariate data. Given there were no material differences in these two approaches, we presented the results based on the full cohort. The proportional hazard assumption was evaluated by assessing interactions between covariates with time and with Schoenfeld residuals; no significant violations were found. We conducted stratified models to determine whether the association between antidepressant use and recurrence risk differed by the initial cancer treatment (surgery; hormonal; radiation; watchful waiting/active surveillance). We also conducted additional sensitivity analyses to assess the robustness of the multivariable results of the association between antidepressant use and biochemical recurrence on the subset of men who had grade codes (proxy of Gleason scores), baseline PSA, and statin and metformin use (*n =* 6,396).

Additionally, we conducted another analysis to examine risk of biochemical recurrence by cumulative duration (total days supplied prior to patients’ earliest study endpoint) in the subset of men who used antidepressants (*n =* 5,931). *P*-values for trend were calculated with the Cochran Mantel–Haenszel test. All analyses were performed using SAS 9.4 (SAS Institute Inc).

## Results

### Demographic and clinical characteristics

In the 10,017 prostate cancer survivors with documented depressive disorders, 5931 (59%) used antidepressants during study follow-up (Table 1). Records of depressive disorder diagnoses were mainly found after prostate cancer diagnosis (96%), and only 4% of the men had depression disorder at baseline (at time of prostate cancer diagnosis and up to one year prior). Among men aged 65–80 years at the time of prostate cancer diagnosis, 62% used antidepressant medication compared to 55% in those aged 30–64 years (*P <* 0.0001). Men of color (i.e., Hispanic men [53%] and African American men [56%]) were less likely to use antidepressant medication compared to non-Hispanic white men (62%) (*P =* 0.0001). We found no significant differences in the antidepressant use by geocoded median household income (*P <* 0.09), nor by anxiety status, or at the time of prostate cancer diagnosis (*p =* 0.25). Men who used antidepressants were more likely to have been diagnosed with stage II disease (60%) compared with stage I disease (54%) and undergo prostatectomy, compared to men who did not use antidepressants, respectively (*P <* 0.05 for all variables). Antidepressant medication users were also more likely to be current smokers (66%) compared to non-antidepressant users (34%), as well as report alcohol misuse (*P <* 0.001 for both variables). Men who used antidepressants had a higher Charlson Comorbidity Index (*P =* 0.001), more likely to have had surgery or radiation (*P* < 0.001), and had a higher median annualized numbers of outpatient visits (17 visits, interquartile range [IQR]: 11–25 visits) compared with non-antidepressant users (15 visits, IQR: 10–23 visits) (*p <* 0.001).

**Table 1:** Demographics and clinical characteristics of prostate cancer survivors with depressive disorders by antidepressant use (*n =* 10,017)

	Antidepressants		No Antidepressants		*P-*value	Total	
	*n =* 5,931		*n =* 4,086			*n =* 10,017	
	N	%	N	%		N	%
Age at Prostate Cancer Dx (yrs)					< .0001		
30–49	199	3.4	188	4.6		387	3.9
50–64	2,201	37.1	1,738	42.5		3,939	39.3
65–80	3,083	52.0	1,876	45.9		4,959	49.5
80 +	448	7.6	284	7.0		732	7.3
Race/Ethnicity					< .0001		
Non-Hispanic White	3,885	66.6	2,435	60.8		6,320	64.3
Hispanic	787	13.5	682	17.0		1,469	15.0
African American/Black	878	15.1	689	17.2		1,567	16.0
Asian/Pacific Islander	280	4.8	197	4.2		477	4.9
Other/Unknown	101	n/a	83	n/a		184	n/a
Geocoded Median Household Income					0.09		
Lower 25%	1,364	23.5	1,010	25.2		2,374	24.2
> 25–50%	1,458	25.1	961	24.0		2,419	24.6
> 50–75%	1,463	25.2	1,048	26.2		2,511	25.6
Top 25%	1,531	26.3	985	24.6		2,516	25.6
Unknown/Missing	115	n/a	82	n/a		197	n/a
Anxiety					0.25		
No	5,206	87.8	3,555	87.0		8,761	87.5
Yes	725	12.2	531	13.0		1,256	12.5
Charlson Comorbidity Index					0.001		
0	3,320	56.9	2,381	59.6		5,701	58.0
1 to 2	1,734	29.7	1,142	28.6		2,876	29.3
3 +	785	13.4	470	11.8		1,255	12.7
Unknown/Missing	92	n/a	93	n/a		185	n/a
Body mass index (kg/m^2^)					0.0013		
Underweight (< 18.5)	22	0.7	12	0.5		34	0.6
Healthy (18.5–24.9)	650	19.7	545	22.7		1,195	21.0
Overweight (25.0–29.0)	1,490	45.2	1,052	43.8		2,542	44.6
Obese (> 30.0)	1,136	34.5	792	33.0		1,928	33.8
Unknown/Missing	2,633	n/a	1,685	n/a		4318	n/a
Smoking					< .0001		
Never smoker	2,762	50.4	1,972	55.7		4,734	52.5
Current smoker	646	11.8	326	9.2		972	10.8
Former smoker	2,075	37.8	1,240	35.1		3,315	36.8
Unknown/Missing	448	n/a	548	n/a		996	n/a
Alcohol misuse					< .0001		
No	3,033	56.4	1,903	54.4		4,936	55.6
Yes	2,348	43.6	1,598	45.6		3,946	44.4
Unknown/Missing	550	n/a	585	n/a		1,135	n/a
Stage at PC diagnosis					0.0003		
Stage I	660	11.1	552	13.5		1,212	12.1
Stage II	5,271	88.9	3534	86.5		8,805	87.9
Prostate cancer treatment					< .0001		
Surgery (Prostatectomy)	2,419	40.8	1,531	37.5		3,950	39.4
Hormonal Therapy	556	9.4	420	10.3		976	9.8
Radiation	1,207	20.4	733	18.0		1,940	19.4
Watchful waiting/active surveillance	1,749	29.5	1,402	34.3		3,151	31.5
Year of PCa diagnosis					< .0001		
1996–1999	614	10.4	513	12.6		1,127	11.3
2000–2004	1,696	28.6	965	23.6		2,661	26.6
2005–2009	1,929	32.5	1,260	30.8		3,189	31.8
2010–2014	1,260	21.2	1,008	24.7		2,268	22.6
2015–2017	432	7.3	340	8.3		772	7.7
Annualized no. of outpatient visits					< .0001		
Median (IQR)	16.9	11.3–25.4	14.9	9.6–22.8			

	Antidepressants		No Antidepressants		*P-value *	Total	
Subset*	*n =* 3,764		*n =* 2,632			*n =* 6,396	
	N	%	N	%		N	%
PSA baseline (quartiles)					< 0.502		
Lower 25%	981	26.1	713	27.1		1,694	26.5
> 25–50%	965	25.6	682	25.9		1,647	25.8
> 50–75%	915	24.3	647	24.6		1,562	24.5
Top 25%	903	24.0	590	22.4		1,493	23.4
Grade group^a^					0.29		
1	2,062	54.8	1,455	55.3		3,517	55.0
2	1,322	35.1	942	35.8		2,264	35.4
3	380	10.1	235	8.9		615	9.6
Metformin					< .0001		
Yes	516	13.7	260	9.9		776	12.1
No	3,248	86.3	2,372	90.1		5,620	87.9
Statins					< .0001		
Yes	2,024	53.8	1,203	45.7		3,227	50.5
No	1,740	46.2	1,429	54.3		3,169	49.5

*Based on subset of *n =* 6,396 subjects with information on these variables: Grade group/Gleason score; PSA at baseline; metformin and statin use

^a^Grade group maps to Gleason scores: Grade code 1 = Gleason sore ≤ 6; Grade code 2 = Gleason score 7; Grade code 3 = Gleason score 8–10

Table 2 presents the distribution of antidepressant classes by the number of prescriptions and number of men who used antidepressants during study follow-up (*n =* 5931). As expected, the most common class were SSRIs (46%) followed TCA (22%) when examining the distribution by prescription type (left panel), and patients used multiple classes of antidepressants (right panel); thus, the total percent exceed 100% for both units of measurement. Overall, the mean cumulative duration of any antidepressant use was 2.42 years (median:1.32 years; IQR: 200 days-3.3 years).

**Table 2:** Distribution of psychiatric drugs use in the subset of prostate cancer survivors treated with such medications during follow-up (*n =* 5931 men)

	N	%	N**	%**
	Unit of observation = prescription		Unit of observation = subject	
Anti-anxiety drugs*	5,999	6.6	1,046	17.6
Antidepressants				
Monoamine oxidase inhibitors	360	0.4	32	0.5
Norepinephrine dopamine reuptake inhibitor/Norepinephrine reuptake inhibitor	8,551	9.3	962	16.2
Serotonin antagonist and reuptake inhibitors	89	0.1	14	0.2
Serotonin modulator and stimulator	144	0.2	8	0.1
Serotonin and norepinephrine reuptake inhibitors	6,704	7.3	834	14.1
Selective serotonin reuptake inhibitors	41,652	45.5	3,623	61.1
Tricyclic antidepressants	20,112	22.0	2,679	45.2
Tetracyclic antidepressants	4,835	5.3	707	11.9
Other	3,113	3.4	406	6.9
TOTAL	91,559	100.0	10,311	173.9

*Alprazolam; chlordiazepoxide; clonazepam; diazepam; lorazepam

**Not mutually exclusive; exceeds 100%

The cohort was followed a maximum of 22 years (median: 6.2 years; IQR: 2.8–10.5 years). Over the 69,500 person-years of follow-up, a total of 1842 men developed biochemical recurrence. The biochemical recurrence rate was higher for antidepressant non-use: 31.3/1000 person-years, compared to antidepressant use: 23.5/1000 person-years (Table 3).

**Table 3:** Biochemical recurrence in 10,017 prostate cancer survivors by antidepressant use

	Biochemical recurrence (N)	Person-years	Rate per 1,000 PYs
All men (*n =* 10,017)			
Antidepressant use	992	42,311	23.45
Antidepressant non-use	850	27,191	31.26

In the multivariable adjusted Cox proportional hazards model, treating antidepressant use as time-varying, the biochemical recurrence risk for antidepressant non-use was 34% higher (adjusted HR = 1.34, 95% CI: 1.24–1.44) than for antidepressant use after adjusting for age; stage and year of prostate cancer diagnosis; baseline PSA; Gleason score; race/ethnicity; geocoded median household income; comorbidity status; body mass index; anxiety history; use of anti-anxiety medications; smoking and alcohol misuse; statin and metformin use; and annualized outpatient office visits (Table 4). The increased risk of biochemical recurrence persisted even after stratifying the results by type of initial prostate cancer treatment. For example, in men who underwent surgery, the biochemical recurrence risk was 56% higher for antidepressant non-use compared to use (adjusted HR = 1.56, 95% CI: 1.34–1.81). In men who selected watchful waiting/active surveillance, the biochemical recurrence risk was 23% greater for antidepressant non-use compared to use (adjusted HR = 1.23, 95% CI: 1.08–1.39). In those treated with hormonal therapy (androgen deprivation therapy), we observed a 38% higher risk (adjusted HR = 1.38, 95% CI: 1.19–1.) for antidepressant non-use compared to use.

**Table 4:** Overall and adjusted risk of prostate cancer progression by prostate cancer treatment and antidepressant use

All men (*n =* 10,017)	Overall HR			Adjusted HR		
	HR	95% CI		HR*	95% CI	
Antidepressant use	1.00	(ref)		1.00	(ref)	
Antidepressant non-use	1.14	1.06	1.22	1.34	1.24	1.44
Surgery (*n =* 3950)						
Antidepressant use	1.00	(ref)		1.00	(ref)	
Antidepressant non-use	1.27	1.10	1.46	1.56	1.34	1.81
Hormonal (*n =* 976)						
Antidepressant use	1.00	(ref)		1.00	(ref)	
Antidepressant non-use	1.14	1.00	1.31	1.38	1.19	1.60
Radiation (*n =* 1940)						
Antidepressant use	1.00	(ref)		1.00	(ref)	
Antidepressant non-use	1.17	1.01	1.36	1.26	1.07	1.47
Watchful waiting/Active surveillance (*n =* 3151)						
Antidepressant use	1.00	(ref)		1.00	(ref)	
Antidepressant non-use	1.07	0.95	1.20	1.23	1.08	1.39

*Adjusted for all variables in Table 1 including medical center

In a subset of *n =* 6396 men with known grade group (proxy for Gleason score), baseline PSA, statin and metformin use, the hazards ratio (adjusted HR = 1.38, 95% CI: 1.27–1.51) was similar to that of the full cohort based on *n =* 10,017 men (adjusted HR = 1.34, 95% CI: 1.24–1.44), again suggesting that antidepressant non-use was still associated with an increased risk of biochemical recurrence/progression risk (data not shown).

In another analysis, we examined the risk of biochemical recurrence by cumulative duration of antidepressant use in the subset of *n =* 5931 men exposed to such medications (Table 5). The risk of biochemical recurrence decreased with longer duration (days supplied) of antidepressant use; for example, compared to those who used antidepressants ≤ 1 year, the risk of biochemical was 67% lower in those who used antidepressants > 3 years (adjusted HR = 0.33 [0.27–0.40]) even after adjustment for the aforementioned covariates. Moreover, the test for trend by number of years of use was statistically significant, *P <* 0.001.

**Table 5:** Risk of biochemical recurrence by cumulative duration of antidepressant use in men treated with antidepressants (*n =* 5931 subset)

	Biochemical recurrence	No recurrence	Crude	Adjusted
Cumulative duration of antidepressant use	*n =* 992	*n=* 4939	HR (95% CI)	HR (95% CI)*
< 1 year	483	2016	1.00 (ref)	1.00 (ref)
> 1–2 years	254	894	1.21 (1.04–1.44)	1.09 (0.93- 1.27)
> 2–3 years	108	550	0.79 (0.64–0.97)	0.70 (0.57- 0.87)
> 3 years	147	1479	0.34 (0.28–0.41)	0.33 (0.27- 0.40)
	*P* test for trend < 0.001			

*Adjusted for variables listed in Table 1

## Discussion

In the diverse cohort of 10,017 prostate cancer patients cared with documented depressive disorders followed over 20 years, the overall rate of prostate cancer biochemical recurrence was higher for antidepressant non-use (31.2/1000 person-years) compared to antidepressant use (23.5/1000 person-years). This corresponded to a 34% higher biochemical recurrence risk for antidepressant non-use compared to use (adjusted HR = 1.34, 95% CI:1.24–1.44). Further, this increased risk was observed in all prostate cancer primary treatment groups (surgery, radiation, hormonal, and watchful waiting/active surveillance) even after accounting for demographics, comorbidity status, tumor characteristics, lifestyle variables, and healthcare utilization. Moreover, the biochemical recurrence risk decreased by longer cumulative duration of antidepressant use in the subset of men exposed to such medications.

These findings have public health implications and demonstrate that prostate cancer survivors should be prioritized for depression screening and treatment of depressive disorders, given that early recognition and treatment has the potential to influence prostate cancer recurrence risk. Furthermore, given that adherence to antidepressant medications is frequently poor, further research on whether monitoring adherence can potentially affect prostate cancer recurrence is needed. Dissemination of the favorable influence of antidepressant medications on prostate cancer recurrence, as well as depression outcomes, has the potential to increase clinician and patient awareness and improve adherence to antidepressant medication treatment.

A potential mechanism for our findings is that depression may increase biochemical recurrence risk through behavioral changes. For example, men with depression are at a greater risk of non-adherence to prescribed medications or healthcare provider treatment recommendations, or they may engage in fewer positive health behaviors [18]. Thus, these behavioral changes can lead to significant depression-associated morbidity (e.g., decreased treatment effectiveness, increased number of cancer-related complications, and/or the development of additional comorbidities [19–24] and mortality, which may also accelerate biochemical recurrence. Depression is associated with poor diet and lower physical activity, which are also risk factors for biochemical recurrence [25]. In all, the high depression prevalence among men diagnosed with early stage prostate cancer, and the negative consequent behavioral changes, warrants further examination of depression as a possible modifiable risk factor for altering prostate cancer outcomes.

Additionally, proposed biologic pathways underlying prostate cancer biochemical recurrence are similar to molecular changes associated with depression, and include chronic inflammation [24, 26–31]; DNA damage [32–37]; telomere shortening [38–42]; and genomic and epigenetic alterations [43, 44]. Combined, this indirectly suggests that depression treatment with antidepressants (which reduce pro-inflammatory cytokines) may affect cancer recurrence risk and warrants further investigation with clinical data.

Our study has several strengths. The maximum study follow-up was over 20 years, and the cohort was diverse; 35% of the group included African American/Black, Hispanic or Asian/Pacific Islander men similar to California’s distribution. This enhances the study’s generalizability. Importantly, we conducted additional analyses based on initial prostate cancer treatment groups to address the fact that antidepressant use could make more of a difference for patients on active surveillance/watchful waiting where depression might affect their compliance with cancer surveillance more so than for patients who underwent surgery. Further, the analysis based on the subset of *n =* 5931 men who used antidepressants demonstrated that longer cumulative duration of antidepressant use was associated with lower risk of biochemical recurrence; this enhances the biologic plausibility of the association. Study medication use was extracted from pharmacy dispensing records mitigating recall bias. Further, patients had similar healthcare access in this managed care organization, and therefore, bias resulting from variable medical coverage was reduced. We were able to account for multiple covariates rarely accounted for in prior studies, such as cancer treatments, tumor characteristics, comorbidity, and sociodemographics, which were captured from electronic health records. We also adjusted for annualized outpatient office visits because men with more clinic visits might have had a greater likelihood of being diagnosed with biochemical recurrence or depressive disorders. Also, we handled pharmacy data as time-dependent variables in the main multivariable model to address immortal time bias. All these features enhanced our study design.

Certain limitations also need consideration. Although this was an observational study, the cohort was longitudinally followed a maximum of 22 years, and we considered a comprehensive set of covariates. Thus, unlike randomized clinical trials that are susceptible to disenrollment, we were able to track patients for a long period. While other classifications for prostate cancer recurrence exist, we selected definitions based on the AUA and ASTRO which were employed in other major urologic studies [14, 15]. Further, although we used the AUA’s definition of biochemical recurrence that did not distinguish between rising PSA or additional treatment, we cannot fully hypothesize on the mechanisms how antidepressant use and depression influence prostate cancer outcomes; however, our application of this definition of biochemical recurrence has been applied in several publications [45–52]. Additionally, because we did not find records on depression severity nor on psychotherapy (“talk therapy”) utilization in this health plan, we cannot address if or behavioral health interventions may also reduce the possible depression-induced risk of biochemical recurrence. Another limitation is that we could not examine the biochemical risk by individual antidepressant drug classes due to the potential low numbers of recurrences for some of these classes. Even larger cohorts are needed to confirm the individual effects of the antidepressants, and if the association between non-use of antidepressants and biochemical recurrence risk is stronger in men with more severe depressive disorders. Residual confounding from physical activity is possible; however, we controlled for body mass index, which may be a proxy. Further, studying effect of various combinations of the nine types of antidepressants, their heterogeneous biologic mechanisms, and mechanisms following drug switching was beyond the scope of this study.

In summary, only 60% of prostate cancer survivors with documented depressive symptoms received antidepressant therapy in this managed care system; this is consistent with a recent meta-analysis that determined half of cancer patients who screen positive for depressive symptoms undergo pharmacologic treatment [53]. Further, this is the first large population-based observational study to suggest that untreated depressive disorders in prostate cancer survivors is associated with an increased risk of biochemical recurrence. Antidepressant medications are not appropriate for all patients. Even though both pharmaceutical and non-pharmacological treatments are available, antidepressants are used more frequently than psychological interventions given inadequate resources in managed care organizations. Notwithstanding the study’s limitations, our findings highlight that depression screening is needed as a part of cancer survivorship care plans given that early identification of depression and its treatment (via medications, psychotherapy, or other behavioral interventions) has the potential to improve both depression and cancer outcomes.

## Data Availability

The study datasets are available upon reasonable request. De-identified data are available from the corresponding author. Data use agreements may be required.
